# Notes and Notices

**Published:** 1899-09

**Authors:** 


### NOTES AND NOTICES.

DR. E. S. BEAUMONT has removed to Luling, Texas. His former address was San Antonio, Texas. The HOMŒOPATHIC PHYSICIAN extends to him greeting and best wishes for success.

THOUSANDS UPON THOUSANDS of people in both Europe and America are being restored to health and strength by the use of Speer's Port Wine. This Wine is a little higher in price but worth five times as much as others for invalids on account of the iron derived from the soil of the extensive vineyards in which the Oporto Grape vines from Portugal are grown in New Jersey. Speer's Port Wine has been adopted by the most scientific and experienced physicians.

DR. WILLIAM DAVIS FOSTER, 420 West Eleventh Street, will remove his office to the Altman Building, southeast corner of Eleventh and Walnut Streets, Room 522, about September 15th. Office hours 2 to 5 P. M. Telephone, Main, 2995.

GRAPES OVERHANG TWO MILES OF CARRIAGE DRIVES.—Grape Arbors loaded with Grapes two miles long and over 300 miles of vines trained on wires. This is the extent of Speer's Oporto Grape Vineyard at Passaic, N. J., only twelve miles from New York City. Those who doubt it can have their expenses paid and $100 given them by the Speer N. J. Wine Co., if they will come and see and do not find the above true.

A Good Thing Mother was at Home.—The *Journal of Medicine and Science* for August quotes the following from the *Sanitarian:*

"Well, Maggie," asked a teacher of a little girl, "how is it you are so late this morning to school?"

"Please, sir, was the reply, "there was a wee bairn cam' to oor hoose this mornin'."

"Ah!" said the teacher, with a smile; "and wasn't your father very pleased with the new baby?"

"No, sir; my father's awa' in Edinburgh, and dinna ken aboot it yet; but it was a good thing my mither was at hame; for gin she had been awa,' I wadna hae kent what to dae wi' it."

Exemption from Testifying.—Governor Roosevelt has signed an amendment to the Civil Code which prohibits absolutely a physician from divulging any information concerning one of his patients, either before or after the death of the latter. Up to the present time the insurance law has permitted the physician to testify concerning the physical condition of the policyholder.—*Medical Times.*

The Bubonic Plague.—The Russian health authorities are reported to be alarmed at the advance of the plague towards Europe. It appeared some time ago in Russian Turkestan and is now said to be raging in Mecca.—*Medical Times.*

Leprosy.—A bill has been introduced into Congress asking that a commission of medical officers of the marine hospital service be formed to investigate the origin and prevalence of leprosy in the United States, and to report upon what legislation is necessary to prevent the spread of the disease.—*Medical Times.*

The eminent surgeon closed up his pocket book with a snap on the $100 fee a wealthy patient had just paid him for a successful operation for appendicitis. "Tell me the appendix vermiformis is a useless organ, will you?" he soliloquized.—*Public Health Journal.*

"Our standing army would be simply rank were it not for the officers."

"There is always a tender connection between the locomotive and the train."

There was an old man named Jerome,
Ate shad and he swallowed a bone.
He cried I shall die.
The doctor said why—
I will call it up with the 'phone.

